# Exposition of a Libel upon the Memory of the Ancients

**Published:** 1819-03

**Authors:** George Frederic Collier

**Affiliations:** Secretary to the Swansea Medico-Chirurgical Society.


*For the London Medical and Physical Journal.*

### *Exposition of a Libel upon the Memory of the Ancients;* by GEORGE FREDERIC COLLIER, Esq. Secretary to the Swansea Medico-Chirurgical Society.

HAVING, from my youth, entertained a considerable degree of veneration for the ancients, and more particularly for Galen and Hippocrates, I was much surprised to hear with what contempt they are spoken of by the different lecturers in the schools of this metropolis. Some degree of indignation was, however, added to that surprise, when I heard them dogmatically assert that the ancients did not know that the arteries contained blood. This assertion, after reading their works, I cannot believe; and, as I am given to understand that this erroneous opinion is very prevalent and inculcated in other schools, I trust it will be a sufficient apology for offering these remarks to the public. Their object is to show, that Galen not only did not believe that the arteries contained air, but likewise that he made experiments to ascertain their structure, which have not been excelled in ingenuity even by Harvey himself.

Though well aware how extremely deficient the ancients were in physiological knowledge, I cannot consent to hear the above accusation brought against them without denying it; considering it as a scandalous libel on their memory.

If we refer to the book which Galen wrote to show "*an sanguis in arteriis natura contineatur*," we shall find him completely master of his subject, and proving to us (to use his own expression) that the "arteries do by nature contain blood, as sure as that fire is hot and snow is cold:" we shall find him clearly demonstrating the absurdity of the contrary opinion entertained in his time by Erasistratus and his followers. Even Erasistratus, however, was not so blind as to suppose that the arteries did not contain blood: his idea was, that they contained blood and a subtle spirit; and we, therefore, learn from Galen, who very closely examines the plausibility of this opinion, that he gravely disputed whether, on making a wound in an artery, the blood or the spirit would first escape. It will not be requisite to make numerous quotations to prove what has been advanced; let the following, therefore, suffice:—

"Ubi funiculo nudatam arteriam utrinque ligavimus et quod in medio comprensum fuerat incidimus, sanguine plenam ipsam esse monstravimus." By this experiment, then, he proves that arteries contain blood, and nothing else; and, after endeavouring to show that they possess a contractile power of their own, he thus proceeds:—"Quibus si etiam unum (experimentum) addidero quod è corporum dissectione collegimus finem dicendi faciam. Est autem id quod dicimus ejusmodi: arteriam unam è magnis et conspicuis quampiam nudabis, primoque pelle remota ipsam ab adjacenti suppositoque corpore tandiu seperare non graveris, quoad funiculum circumdare valeas; deinde secundum longitudinem arteriam incide, calamumque et concavum et pervium in foramen intrude; vel æneam aliquam fistulam quo et vulnus obturetur et sanguis exilire non possit. Quoadusque sic se arteriam habere conspicies, ipsam totam pulsare videbis; cum primum verum obductum laqueum contrahens, arteriæ tunicas calamo obstrinxeris non amplius arteriam ultra laqueum palpitare videbis; etiam si sanguis ad arteriam quæ est ultra laqueum, sicuti prius faciebat, per concavitatem calami feratur. Quod si propterea pulsarent arteriæ, pulsarent etiam tum partes quæ sunt ultra laqueum; sed non pulsarent, igitur perspicuum est, quoniam moveri posse desinunt, *non per spiritum in concavitatibus discurrentem sed ob virtutem intunicas transmissum arterias a corde moveri.*"—Vide Librum GALENI *en Sunguis Arteris*, &c. sec. x. et passim.

Thus, it plainly appears that Galen did know that the arteries contain blood,—nay, that he knew more: that he dissected their coats, ascertained their structure, and has left upon record experiments in proof of their muscularity, which even Harvey confesses himself unable to refute, although of a different opinion. I have been more particular in making this last quotation, because (as far as I know) this experiment is not mentioned by any of the lecturers, and because it is undoubtedly a strong proof of the opinion entertained by one part of the medical world,—viz. "that the arteries are muscular." It will not be necessary to quote other ancient writers to support my argument; Galen's testimony is sufficient, who says that Erasistratus, *et ejus sectatores,* were the only men in his time who believed that the arteries contained any thing but blood. It is not merely in the book above quoted that Galen speaks of the nature of arteries; in his books "de Hippocratis et Platonis dogmatibus," we find a recapitulation of his opinions.

There can be no claim to ingenuity in these observations, for the works of Galen are accessible to all: my only object in writing this paper is to endeavour to correct an error which has arisen from a servile deference to derivation, and to show that it is as unjust and uncharitable in us to accuse the ancients of not understanding the nature of arteries, because the appellation they gave to them is incorrect, as it would be for our posterity to bring the same accusation against us, since we also still retain the same unmeaning term.

Harvey, in the preface to his work "de Motu cordis et Sanguinis," does Galen the justice to acknowledge that he was not ignorant of the structure of arteries; and seems to have paid more attention and deference to his experiments and observations than to those of any other writer. He reproaches the physicians of his time who accused this ancient author of the ignorance above alluded to, and asks, who can be so foolish as to doubt Galen's knowledge of the contents of arteries, when he reads his observation—that "ab una arteria dissecta unius semi hora spatio, totam messam sanguinis ab universo corpore, magna et impetuosa profusione, exhaustam fore."

*St. Paul's Church-Yard;*
*December* 2, 1818.

